# Supplementary material for: Comparative genomics provides new insights into the diversity, physiology, and sexuality of the only industrially exploited tremellomycete: Phaffia rhodozyma
Source: BMC Genomics. 2016 Nov 9;17:901. doi: 10.1186/s12864-016-3244-7 (PMC5103461; doi:10.1186/s12864-016-3244-7)
Supplement: Additional file 6: — List of orphan genes with links to PFAM (related to Additional file 1: Table S1). (ZIP 1428 kb) [file 12864_2016_3244_MOESM6_ESM.zip › PFAM_UNIQUE/G05502_P.html]

#                                                                            --- full sequence --- -------------- this domain -------------   hmm coord   ali coord   env coord
  
# target name        accession   tlen query name           accession   qlen   E-value  score  bias   #  of  c-Evalue  i-Evalue  score  bias  from    to  from    to  from    to  acc description of target
  
#------------------- ---------- ----- -------------------- ---------- ----- --------- ------ ----- --- --- --------- --------- ------ ----- ----- ----- ----- ----- ----- ----- ---- ---------------------
  
G05502\_P             -            167 Gal\_Lectin           PF02140.13    80      0.45    9.5   2.8   1   2   8.7e-05      0.52    9.4   0.7    11    57    68   114    66   128 0.76 - Galactose binding lectin domain  
G05502\_P             -            167 Gal\_Lectin           PF02140.13    80      0.45    9.5   2.8   2   2      0.16   9.4e+02   -1.1   0.1    20    36   141   160   120   165 0.52 - Galactose binding lectin domain
